# From biogenesis to aptasensors: advancements in analysis for tumor-derived extracellular vesicles research

**DOI:** 10.7150/thno.95885

**Published:** 2024-07-02

**Authors:** Gaojian Yang, Zhiyang Li, Rabia Usman, Yuan Liu, Song Li, Zhu Chen, Hui Chen, Yan Deng, Yile Fang, Nongyue He

**Affiliations:** 1 State Key Laboratory of Digital Medical Engineering, School of Biological Science and Medical Engineering, Southeast University, Nanjing 210096, China.; 2 China Hunan Key Laboratory of Biomedical Nanomaterials and Devices, Hunan University of Technology, Zhuzhou 412007, PR China.; 3 Department of Clinical Laboratory, the Affiliated Drum Tower Hospital of Nanjing University Medical School, Nanjing 210008, China.; 4 Institute of Cytology and Genetics, School of Basic Medical Sciences, Hengyang Medical School, University of South China, Hengyang 421001, China.; 5 Institute for Future Sciences, University of South China, Changsha Hunan 410000, China.

**Keywords:** Extracellular Vesicles, Tumor Markers, Aptamers, Detection

## Abstract

Extracellular vesicles (EVs) are enclosed by a nanoscale phospholipid bilayer membrane and typically range in size from 30 to 200 nm. They contain a high concentration of specific proteins, nucleic acids, and lipids, reflecting but not identical to the composition of the parent cell. The inherent characteristics and variety of EVs give them extensive and unique advantages in the field of cancer identification and treatment. Recently, EVs have been recognized as potential tumor markers for the detection of cancer. Aptamers, which are molecules of single-stranded DNA or RNA, demonstrate remarkable specificity and affinity for their targets by adopting distinct tertiary structures. Aptamers offer various advantages over their protein counterparts, such as reduced immunogenicity, the ability for convenient large-scale synthesis, and straightforward chemical modification. In this review, we summarized EVs biogenesis, sample collection, isolation, storage and characterization, and finally provided a comprehensive survey of analysis techniques for EVs detection that are based on aptamers.

## Introduction

Cells release extracellular vesicles (EVs) into the extracellular space; these are small, membrane-bound structures composed of a lipid bilayer. EVs facilitate the transfer of various molecules, including proteins, nucleic acids, and lipids, between cells 1, 2. Based on their origin and size, EVs typically range from 30 to 200 nm 3, 4. These vesicles provide critical insights into the health of donor cells and are involved in a wide array of physiological and pathological processes, such as tumor development, immune responses, signal transduction, and antigen presentation 5. Furthermore, EVs are pivotal for the diagnosis and monitoring of diseases, being detectable in bodily fluids like blood, urine, saliva, and cerebrospinal fluid, which highlights their potential as noninvasive biomarkers 6-8. Analyzing EVs is challenging due to their small size; however, employing antibodies that target specific protein markers on the EV surface facilitates their visualization and quantification. This approach allows for both qualitative and quantitative assessments of vesicular protein markers 9-11. Nevertheless, the glycosylation of proteins on EVs can impede the binding of fluorescently labeled antibodies, thus reducing detection sensitivity 12.

Aptamers are short, single-stranded nucleic acids (DNA or RNA) that can bind to specific target molecules with high affinity and specificity. Often referred to as the nucleic acid counterparts of antibodies, aptamers are synthesized through a method known as Systematic Evolution of Ligands by Exponential enrichment (SELEX). This process involves iterative cycles of selection and amplification to isolate aptamers that exhibit the desired binding properties 13. Aptamers offer numerous benefits over conventional antibodies, including their smaller size, ease of synthesis, and chemical stability 14, 15. In the context of EVs, aptamers can be tailored to selectively recognize and bind to exosomal proteins or other surface markers, facilitating the development of aptasensors. These biosensors employ aptamers as the recognition element and enable the construction of compact, on-site devices for the detection of EVs 16. Aptamers are characterized by their high specificity, ease of synthesis & modification, excellent stability, low immunogenicity, and minimal toxicity. They can also be seamlessly integrated into various sensor systems, enhancing the development of portable devices for immediate EVs identification.

**Figure 1:**
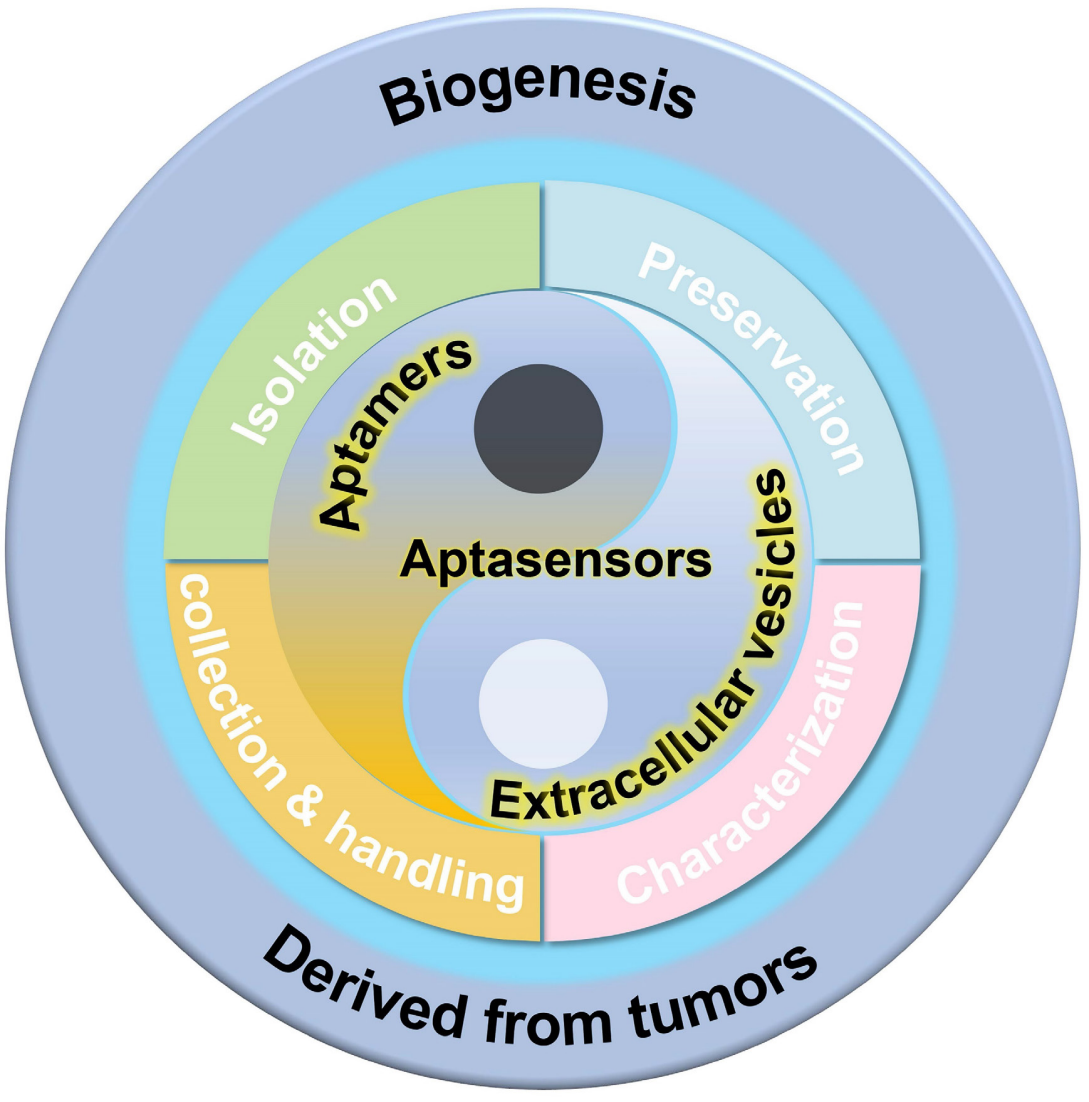
Schematic illustration of analysis for tumor-derived extracellular vesicles, from biogenesis to the development of aptasensors.

In this review, we aim to provide a comprehensive analysis of recent progress in the methodologies for studying EVs. The review explores advancements in several domains, including the production of EVs, sample collection, separation, preservation, and analysis, as illustrated in Figure 1. Additionally, we examine a broad spectrum of aptamer-based detection techniques that employ diverse signaling modalities, such as optical, electrical, and so on.

## From Biogenesis to Characterization of Extracellular Vesicles

### Biogenesis of EVs

**Figure 2:**
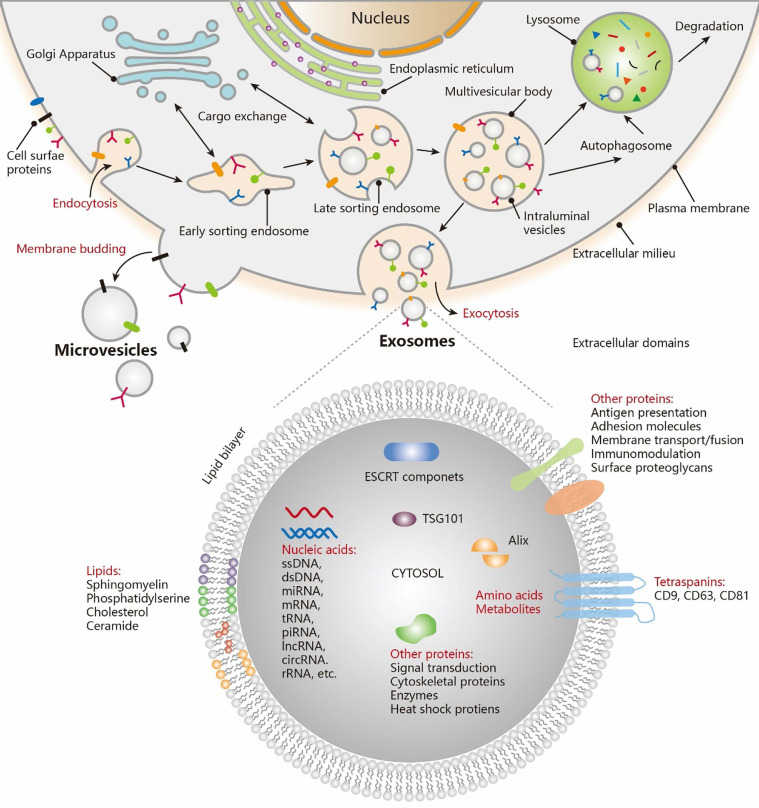
Biogenesis and contents of EVs. Exosomes are generated via the double invagination of the PM, including the formation of early sorting endosomes (ESEs) and late sorting endosomes (LSEs), the generation of ILVs within MVBs, the transportation of MVBs to cytoplasmic membrane, and the fusion of MVBs membrane with the cell membrane. Cargo exchange occurs between ESEs (LSEs) and the trans-Golgi network. MVBs can also fuse with lysosomes or autophagosomes for the degradation and recycling of cellular contents. Microvesicles are derived from the direct outward budding of cellular PM. EVs, including exosomes, transport a vast repertoire of different types of proteins, lipids, NAs, and other small molecules. Adapted with permission from 22, copyright 2021 American Chemical Society.

The existence of membrane-structured microvesicles, later named "exosomes," was first demonstrated in maturing erythrocytes in 1983 17, and the term "exosome" was officially adopted in 1987 18. Exosomes, a distinct subtype of EVs, are typically small in size. Numerous studies have shown that cellular communication can occur through the release of EVs 19-21. EVs are produced through the cellular endocytosis process, carrying intracellular substances, and are prevalent in various body fluids. They play a pivotal role in facilitating intercellular communication, transporting cargo, and regulating diverse pathophysiological processes. The traditional view holds that endocytosis leads to the formation of intraluminal vesicles (ILVs), which merge with the endosome membrane. These endosomes then evolve into multivesicular bodies (MVBs), incorporating "cargo" such as RNA, proteins, and lipids from the cytoplasm (Figure 2 22). EVs are expelled through the inward budding of the limiting membrane of MVBs as early endosomes mature into late endosomes 23-26. However, an alternative theory suggests that the plasma membrane can also directly release EVs through vesicle budding or gradually release them into the extracellular matrix via budding at intracellular plasma membrane-connected compartments 27-29. Both theories agree that vesicles (ranging from 30-200 nm) are produced from endosomes and plasma membranes through budding. The ILVs are released into the extracellular environment upon the fusion of MVBs with cellular membranes. The primary distinction lies in whether vesicles derived from the plasma membrane should be classified as EVs.

### Sample collection and handling

**Figure 3:**
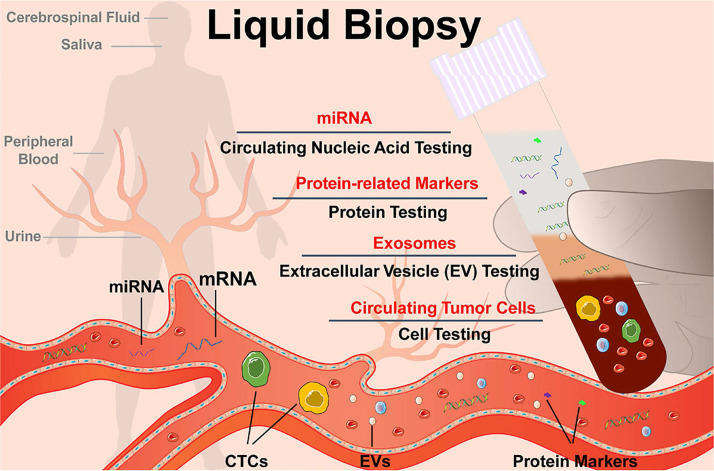
Schematic diagram of liquid biopsy. Adapted with permission from 30, copyright 2021 American Chemical Society.

Research has focused on developing novel techniques for the preparation and processing of EVs samples. A variety of physical principles, functional compounds, and engineering methods have been employed to improve the characterization of EVs and associated biomolecules. However, several challenges still need to be addressed in the future. One such challenge is the requirement for distinct processing procedures for EV samples derived from different body fluids (Figure 3) 30. EVs are demonstrated that presented in almost all the body fluids including cell culture-conditioned media 31-35, blood/serum/plasma 36-40, pleural effusions and ascites 41-44, saliva 45-48, urine 49-55, tear fluid 56-59, and cerebrospinal fluid (CSF) 60-63. The concentration, composition, and bioactivities of EVs can vary significantly depending on the type of body fluid, collection strategies, storage conditions, and pretreatment methods 64, 65. Several biological sources of EVs are commonly investigated. A summary of sample collection and handling is presented in Table 1.

### Isolation of EVs

**Figure 4:**
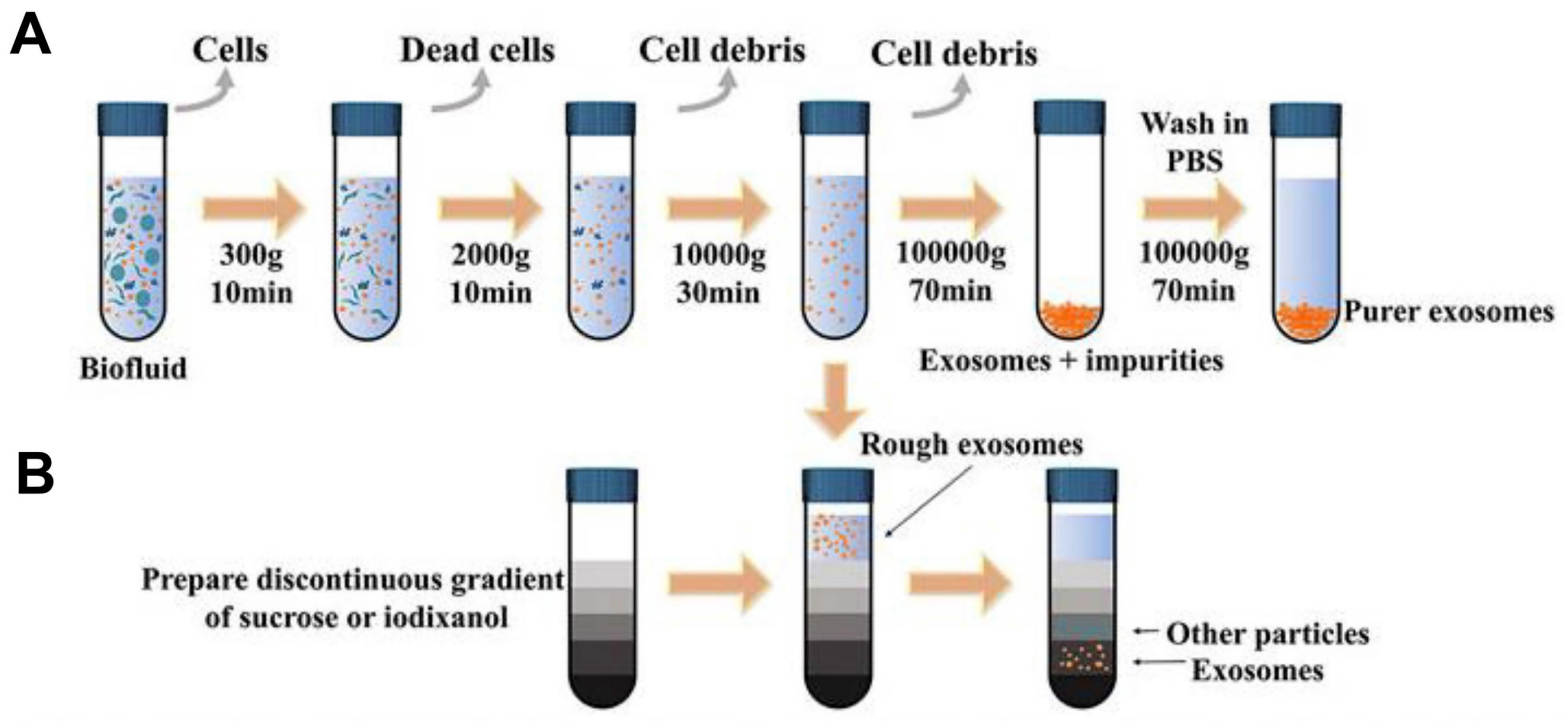
Schematic representation of common exosomal separation techniques. (**A**) Ultracentrifugation, (**B**) Density gradient centrifugation. Adapted with permission from 69, copyright 2022 Frontiers.

EVs play a crucial role in facilitating information and content exchange between different cells, which significantly impacts the early diagnosis and treatment of tumors. High recovery and purity in separation are essential first steps for EVs -related detection and analysis 66-68. Various isolation techniques were classified on the basis of the methodology and properties of EVs: (1) ultracentrifugation-based isolation strategies (Figure 4) 69, 70, (2) size-based isolation strategies 71, 72, (3) precipitate and phase separation 73, (4) membrane-based isolation 74, (5) microfluidic analysis 75. A summary of EVs isolation strategies is presented in Table 2.

### Preservation strategies of EVs

It is imperative to conduct the subsequent identification, detection, and analysis of EVs promptly due to their short half-life, which can lead to rapid and irreversible degradation 76-79. Preservation strategies for EVs are designed to maintain their bioactivities and facilitate transportation. Techniques such as cryopreservation 80-83, freeze-drying 84-86 and spray-drying 87, 88 have been widely applied in various studies and clinical procedures recently. A summary of EVs preservation strategies is revealed in Table 3.

### Characterization of EVs

The properties of EVs including size, morphology, and components (proteins, RNA and lipid bilayer) are able to distinguish EVs from others EVs, which is the basis of various EVs characterization methods. In this section, as shown in Table 4, we will discuss various qualitative and quantitative methods commonly used for EVs characterization: (1) western blot (WB) 89, (2) microscopy-based characterization 90-92, (3) nanoparticle tracking analysis (NTA) 93 and dynamic light scattering (DLS) 94, 95, (4) flow cytometry (FCM) 96.

## Advantages of aptamers as detection probes

**Figure 5:**
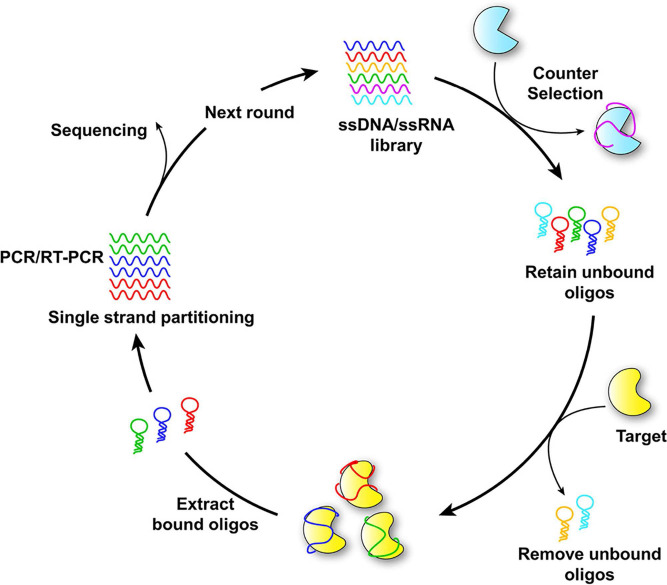
Schematic diagram of SELEX for aptamer selection. Adapted with permission from 30, copyright 2021 American Chemical Society.

Aptamers, brief single-stranded DNA or RNA molecules, can bind to specific target molecules with high affinity and specificity. Due to their ability to recognize and bind to a variety of targets, including proteins, peptides, small molecules, and even entire cells, they are often referred to as "chemical antibodies" 97. The SELEX process generates aptamers through iterative rounds of selection and amplification, aiming to identify those with high affinity for specific target molecules. Techniques such as surface plasmon resonance (SPR), fluorescence-based assays, and isothermal titration calorimetry (ITC) are utilized to evaluate the binding affinity, specificity, and stability of selected aptamers. Based on the results of analysis and characterization, aptamers can be further refined through rational design or mutagenesis to enhance their binding properties, stability, or other desired characteristics. The principle of aptamer selection by SELEX is illustrated in Figure 5 30.

In diagnostics, aptamers serve as tools for the detection and quantification of target molecules, including cancer biomarkers, pathogens, and toxins. In therapeutics, aptamers are developed as targeted drug delivery vehicles or as therapeutic agents themselves, capable of inhibiting or modulating the activity of specific proteins 64, 98. Aptamers have the potential to revolutionize the field of molecular recognition and offer new opportunities for the development of innovative analytical technologies 99. Aptamers offer several advantages when used as probes in EVs detection: (1) High affinity and specificity: SELEX facilitates the selection of aptamers with high affinity and specificity for targets, enabling accurate and reliable detection of target molecules 15. (2) Versatility: Aptamers can target a diverse array of entities, including small compounds, proteins, EVs, and cells. This adaptability makes them suitable for detecting various substances across different biological specimens 100. (3) Batch-to-batch consistency: Aptamers can be synthesized with high reproducibility, ensuring consistent performance across different batches, crucial for diagnostic applications where reliable results are paramount 101. (4) Ease of modification: Aptamers can be readily modified with various fluorescent labels or other signaling molecules, facilitating the integration of diverse detection methods. This flexibility allows for the development of sensitive assays capable of detecting multiple targets 102. (5) Enhanced penetration and sensitivity: Due to their smaller size compared to antibodies, aptamers achieve better tissue and cellular penetration, improving detection sensitivity, particularly when the target molecule is in hard-to-reach areas 103. (6) Reusability: Unlike antibodies, aptamers can be regenerated and reused multiple times without diminishing their binding affinity or specificity, making them ideal for continuous monitoring or repeated measurements 104. (7) Lower immunogenicity: As synthetic molecules, aptamers do not provoke the same immune response as antibodies, minimizing the risk of adverse immune reactions or interference in diagnostic assays 105. Overall, the unique properties of aptamers position them as valuable tools for developing precise and accurate detection systems in cancer diagnosis and other biomedical applications.

## Detection of EVs

The critical role of EVs is their capacity to facilitate intercellular communication by transporting various biomolecules, such as proteins, nucleic acids, and lipids, between cells. The detection and characterization of EVs have emerged as areas of increasing interest, given their potential as invaluable diagnostic and therapeutic tools. However, the small size and low abundance of EVs in biological fluids present significant challenges for their detection. These challenges have been addressed through recent advancements in analytical technologies, including: (1) fluorescence detection, (2) luminescence detection, (3) electrochemical detection, (4) colorimetric detection, (5) microfluidics detection, and so on. An evaluation of EVs detection methods is summarized in Table 5.

### Fluorescence detection

The process of fluorescence detection employs fluorescent molecules or dyes that specifically bind to EVs or their surface markers. These fluorescent probes emit light upon stimulation by a specific wavelength, enabling the visualization and quantification of EVs. In this technique, EVs are typically labeled with fluorescent antibodies or aptamers that selectively bind to surface proteins on the EVs. The resulting fluorescent signal from the labeled EVs can be detected using fluorescence microscopy or flow cytometry. The development of fluorescence detection primarily incorporates four strategies: nucleic acid-assisted amplification, enzyme-assisted amplification, nanomaterials-assisted amplification, and direct fluorescence detection.

#### Nucleic acid-assisted amplification detection

Nucleic acid-assisted amplification aims to overcome the limitations of low fluorescence intensity exhibited by directly stained EVs and enhances the detection of exosomal protein markers with improved sensitivity. This facilitates more accurate and reliable analysis of EVs in various biological samples. Techniques such as rolling circle amplification (RCA), strand displacement reaction (SDR), hybridization chain reaction (HCR), and catalytic hairpin assembly (CHA) are employed in these processes.

**Figure 6:**
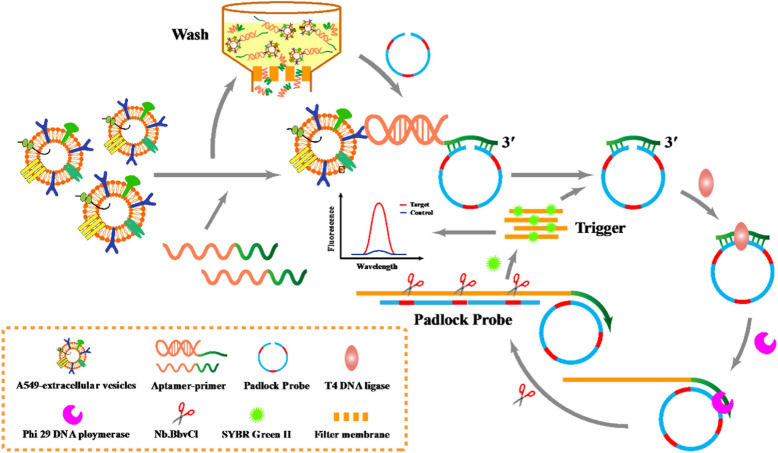
Schematic diagram for P-ERCA. Adapted with permission from 106, copyright 2021 Elsevier.

RCA involves amplifying circular DNA templates using a DNA polymerase with strand displacement activity. The RCA process begins with the hybridization of a circular DNA probe to a specific nucleic acid sequence within the EVs. This probe contains a recognition sequence for DNA polymerase and a primer binding site. Upon hybridization to the target sequence, the DNA polymerase initiates the synthesis of new DNA strands, displacing existing strands and generating a long single-stranded DNA molecule. He *et al.* utilized a padlock probe-based exponential rolling circle amplification (P-ERCA) assay to detect EVs. This method involves conjugating an aptamer targeting the vesicles to a primer sequence, which initiates linear rolling circle amplification in the presence of target EVs 106. Multiple nicking reactions generate trigger DNA, leading to further circle amplification, and the resulting P-ERCA product exhibits an exponential fluorescence signal as shown in Figure 6. This method successfully discriminates lung cancer EVs in real human serum samples. Additionally, a highly specific technique that utilizes branched rolling circle amplification and a padlock probe demonstrated a shorter reaction time (less than 3 h) and a detection limit of 42.7 particles *μ*L^-1^ 107. Similarly, RCA products that employ a CD63 aptamer and DNAzyme achieve targeted identification and amplification of signals within 60 min 108. For clinical diagnosis of urinary diseases, Wu *et al.* described a diagnostic protocol that integrates a ratiometric 3D DNA machine with machine learning techniques, achieving a limit of detection (LOD) of 9.9 particles per microliter and a linear range of 10^4^ to 10^8^ particles *μ*L^-1^ for urinary EVs 109. Moreover, the classification of disease types exhibits accuracies of 94.7% and 89.5%, respectively.

**Figure 7:**
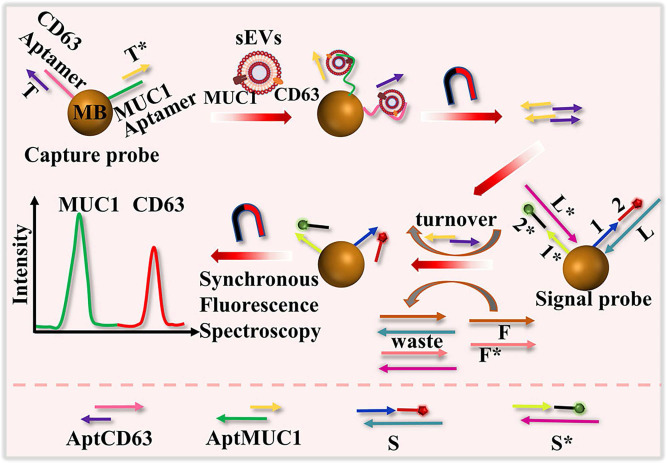
Schematic representation of the detection of surface proteins of sEVs using a dual color DNA nanodevice based on an enzyme-free signal amplification and synchronous fluorescence technique. Adapted with permission from 110, copyright 2022 American Chemical Society.

SDR is a branch of nucleic acid amplification methods that leverages the ability of DNA or RNA strands to displace each other from a target sequence through hybridization and enzymatic activity. This method employs nucleic acid probes that specifically recognize and bind to target molecules on EVs. A DNA nanodevice with dual colors was developed to simultaneously analyze surface proteins (CD63 and MUC1) of EVs using toehold-mediated DNA strand displacement signal amplification and synchronous fluorescence techniques, as illustrated in Figure 7 110. This assay requires 60 min to complete. For more efficient detection, Zhang introduced a fluorescence-based method for analyzing the surface protein of EVs, utilizing an isothermal cascade nucleic acid amplification approach 111. This technique transforms signals from the surface membrane proteins of EVs into nucleic acid signals using a carefully designed capture probe that includes a protein aptamer and a partially complementary blocker. DNA polymerase and Nb.BbvCI nicking enzyme are employed to amplify the signal, allowing the detection process to be completed in just 30 min with a linear range of 10^2^ to 10^6^ particles *μ*L^-1^. Additionally, Chen *et al.* developed a label-free biosensor, selecting N-methylmesoporphyrin IX as the signal reporter. This compound is capable of inserting into the G-quadruplex structure, thereby generating a significant increase in fluorescence 112. SDR exhibits exceptional specificity, as the probe is specifically designed to match the target sequence. Moreover, it demonstrates remarkable sensitivity due to the amplification process, enabling the detection of target sequences present in low quantities. Furthermore, SDR can be conducted in a homogeneous solution, eliminating the need for complex sample preparation procedures.

**Figure 8:**
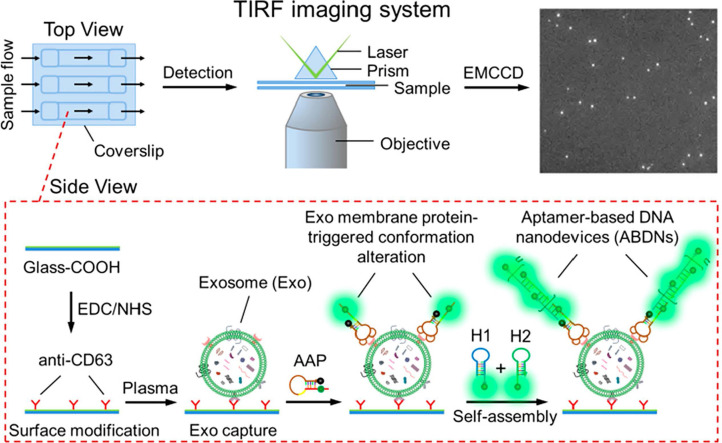
Illustration of the ABDN-based TIRF assay for single-vesicle imaging and detection of circulating tumor-specific Exos in plasma. Adapted with permission from 116, copyright 2019 American Chemical Society.

HCR is a technique for amplifying signals through the combination of two hairpin DNA probes, leading to the formation of an extensive DNA chain. This chain undergoes further amplification by continuously opening and extending the hairpin structures, thus significantly enhancing the fluorescence signal. Zhu *et al.* described a cascade bHCR amplification activated by co-marker recognition, which generates multiple G-quadruplex structures combined with fluorescent dyes to facilitate signal transduction. This method significantly improves accurate detection by effectively minimizing false positives caused by interfering proteins or individual protein markers 113. Additionally, various advanced approaches have been reported, such as employing DNA nanostructures as "nanoweights" to enrich cancerous EVs 114, and utilizing CRISPR-Cas12a assisted HCR 115. For clinical applications, He *et al.* developed a highly sensitive assay that enables direct visualization and measurement of single vesicles in 1 *μ*L plasma, as illustrated in Figure 8 116. This assay depends on the specific interaction between activatable aptamer probes and EVs, which are captured by EVs-specific antibodies affixed to the flow cell's surface, leading to the generation of activated fluorescence. The assay operates within a linear range of 10^3^ to 10^7^ particles *μ*L^-1^.

**Figure 9:**
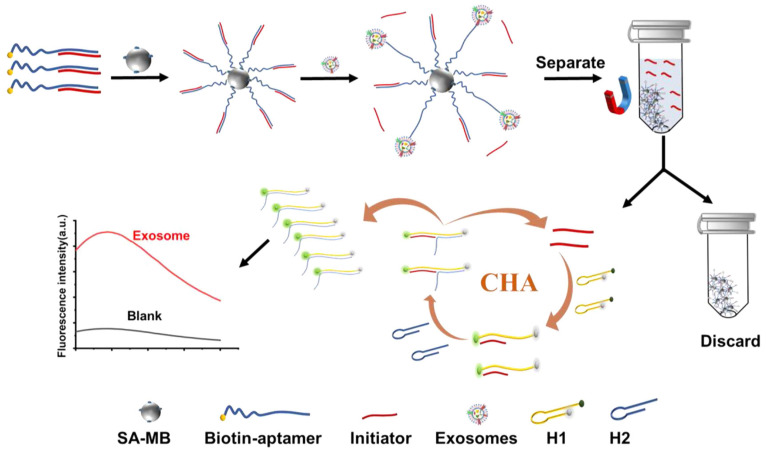
Principle of the aptamer-initiated CHA (AICHA) signal amplification strategy for exosome detection. H1 was modified with a FAM fluorophore and BHQ2 quencher. SA-MB: streptavidin-modified magnetic beads. Biotin-aptamer: biotin modified aptamer. Adapted with permission from 118, copyright 2022 American Chemical Society.

A novel and effective microchip electrophoresis (MCE)-based method has been developed for detecting MCF-7 EVs, utilizing a triple amplification strategy. This approach combines a cholesterol probe (Chol-probe) with displacement amplification-CHA (SDA-CHA) 117. The immobilization of CD63 aptamers on avidin magnetic beads facilitates the specific capture of EVs. Following magnetic separation, the Chol-probe complements ssDNA and initiates SDA, leading to the production of multiple DNA sequences (Ta) that trigger CHA, resulting in SDA-CHA amplification. The LOD for MCF-7 EVs was achieved at an impressive level of 26 particles *μ*L^-1^. Additionally, Zhou *et al.* proposed a robust fluorescence assay based on aptamer-initiated CHA, achieving an LOD of 0.5 particles *μ*L^-1^ for MCF-7 cell-derived EVs and 0.1 particles *μ*L^-1^ for PANC-1 cell-derived EVs, as depicted in Figure 9 118. The assay also demonstrated excellent performance in serum samples, with a recovery rate ranging from 95.45% to 106.2%.

#### Enzymes-assisted amplification detection

DNAzymes are synthetic DNA molecules with enzymatic activity that catalyze specific reactions and can be employed as fluorescence probes for the sensitive and specific identification of EVs. Hao *et al.* developed a DNAzyme assembly featuring an aptamer-bivalent-cholesterol-anchor, which facilitates straightforward identification of exosomal PD-L1 with a LOD of 5.21 pg mL^-1^ 119. This assay is completed in approximately 130 min. Additionally, a sensitive strategy was developed to selectively detect EVs in just 10 min, using multi-component nuclease (MNAzyme) mediated recognition of EpCAM and CD63 proteins on the EV membrane. This strategy enables highly sensitive and specific quantification of EVs in complex biological samples, with an LOD of 72 particles *μ*L^-1^ 120.

The CRISPR/Cas12a system, a molecular scissor, utilizes the Cas12a protein (also known as Cpf1) and a guide RNA (gRNA) to precisely edit targeted DNA sequences by cleaving both strands at specific locations determined by the gRNA. Zhao's group introduced an allosteric probe-initiated dual cycle amplification-assisted CRISPR-Cas12a, demonstrating a direct correlation with isolated EVs across a concentration range of 10^2^ to 10^6^ particles *μ*L^-1^ 121. This approach combines aptamers, PCR-based exponential amplification, and real-time DNA detection using CRISPR/Cas12a. Analysis of clinical serum samples indicated that measuring levels of serum CD109+ and EGFR+ tumor-derived EVs together resulted in high diagnostic accuracy, with an area under the curve (AUC) of 0.934 (95% CI 0.868-1.000), a sensitivity of 84.1%, and a specificity of 85.0% 122. Additionally, biosensors mediated by terminal deoxynucleotidyl transferase (TdT) 123 and restriction endonucleases (Nt. BstNBI) 124 have also been applied for EV detection.

#### Nanomaterials-assisted amplification detection

Nano-materials are engineered to enhance the sensitivity and specificity of detection methods, thereby facilitating the precise determination of EVs levels in biological specimens. Jin *et al.* introduced an EVs -targeted, aptamer nanoprobe-based profiling technique that combines graphene oxide (GO) with aptamers specific to targeted surface proteins, enabling the characterization and quantification of cancerous EVs in a straightforward mix-and-detect format 125. This assay successfully identified the EVs surface protein PSMA in blood samples from prostate cancer patients, demonstrating its potential for clinical diagnostic applications. In a separate study, Xia *et al.* employed the BP@Mn^2+^/DNA hybrid nanosensor for the direct detection of exosomal miRNAs and cancer-specific EVs in clinical plasma samples, aiming to diagnose cancer 126. Alternatively, signal amplification approaches mediated by copper 127 and gold nanoparticles (AuNPs) 128 utilize a sandwich format to enhance sensitivity, specificity, multiplexing capability, non-destructive detection, compatibility with various sample types, and rapid detection. These features make them invaluable tools for EVs analysis in diverse research and clinical contexts.

#### Direct fluorescence detection

Traditional techniques for detecting EVs through fluorescence typically require prior purification and signal amplification steps. However, several studies have demonstrated the feasibility of signal amplification-independent fluorescence detection of EVs with exceptional sensitivity 129, 130. A fluorescence polarization assay utilizing aptamers has been developed, enabling direct quantification of EVs in human plasma without requiring separation or amplification 131. Additionally, to overcome challenges associated with the sensitive and direct detection of EVs from plasma, Wang *et al.* introduced a Single Microbead-based Fluorescent Aptasensor (SMFA). This method combines direct magnetic isolation with in situ fluorescence imaging 132. In this technique, a single microbead (MB) modified with aptamers acts as the reaction carrier, allowing specific EVs with a fluorescent anchor to be highly enriched on the MB. This approach achieves sensitive detection of EVs without requiring signal amplification, by utilizing in situ fluorescence imaging to monitor the signals on the single MB.

### Luminescence detection

Luminescent techniques are employed for the detection and quantification of EVs through light emission. These methods confer advantages such as enhanced sensitivity, specificity, and the capability for real-time analysis. In aptamer-based EVs detection, luminescence is primarily generated through two mechanisms: electrochemiluminescence (ECL) and luminescence resonance energy transfer (LRET).

#### Electrochemiluminescence detection

**Figure 10:**
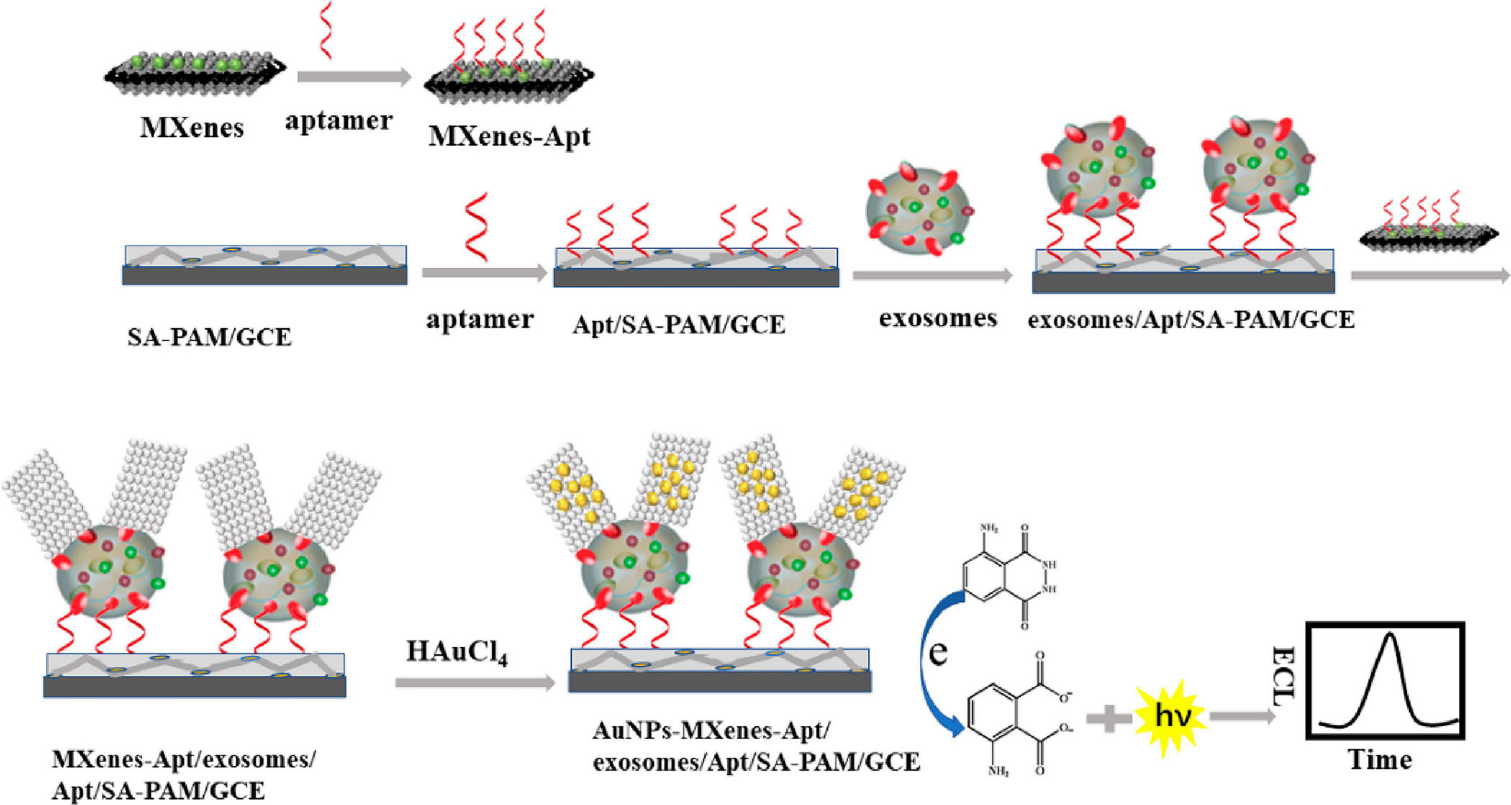
Principle of the ECL biosensor for exosomes detection based on in situ formation of gold nanoparticles decorated Ti_3_C_2_ MXenes nanoprobes. Adapted with permission from 134, copyright 2020 American Chemical Society.

ECL merges the advantages of chemiluminescence and electrochemistry, transforming electrical energy into light emission. This technique provides benefits including low background noise, convenience, rapid response, and high sensitivity. There are two primary strategies for ECL-based EVs detection: signal-enhancement and signal-decrease. The signal-enhancement strategy can be realized through the use of nanomaterials that amplify the ECL signal. Zhang's group developed an ECL biosensor using aptamer-modified Ti_3_C_2_ MXenes nanosheets as the ECL nanoprobe 133. This modification enables efficient capture of EVs on the electrode surface, while the ECL nanoprobe boosts the luminol signals, thereby enhancing EVs detection. Subsequently, they created a highly sensitive ECL biosensor employing a hybrid material composed of AuNPs-decorated Ti_3_C_2_ MXenes with aptamer modifications, as shown in Figure 10 134. This biosensor achieved a detection limit for HeLa cell-derived EVs of 30 particles *μ*L^-1^, significantly surpassing the sensitivity of traditional ELISA methods by over a thousandfold. More recently, Nie *et al.* constructed a self-luminous Faraday cage-type sensing system using a nanoarchitecture that includes a Ti_3_C_2_Tx-Bi_2_S_3_-x heterostructure and an engineered lipid layer 135. This sensor detects tumor EVs in gastric cancer patient ascites without additional purification. Conversely, the signal-decrease strategy involves inhibiting electron transfer, leading to a decrease in ECL signal proportional to the concentration of EVs on the electrode, thus enabling quantitative detection. Qiao *et al.* introduced an ECL aptasensor utilizing mercaptopropionic acid (MPA)-modified Eu^3+^-doped CdS nanocrystals as emitters 136. The CD63 aptamer captures EVs, which then transform into a G-quadruplex/hemin DNAzyme. This DNAzyme accelerates the decomposition of H_2_O_2_, consequently reducing the ECL signal.

#### Luminescence resonance energy transfer detection

LRET is a process where energy is non-radiatively transferred from an excited donor molecule to an acceptor molecule via dipole-dipole interactions across significant distances. This method facilitates the detection of EVs in complex biological matrices while effectively reducing background signal interference. A straightforward aptasensor employing the LRET mechanism between upconversion nanoparticles (UCNPs) and gold nanorods (Au NRs) was developed 137. The extent of luminescence quenching in UCNPs is directly proportional to the EV concentration, which ranges from 1×10^4^ to 1×10^8^ particles *μ*L^-1^, enabling both detection and quantification of EVs. Additionally, Wang *et al.* introduced an aptasensor that operates without washing, using LRET between rare-earth-doped UCNPs as the emitter and tetramethyl rhodamine (TAMRA) as the acceptor 138. In the presence of EVs, TAMRA emits yellow fluorescence at 585 nm due to LRET when the UCNPs-TAMRA system is excited by near-infrared light at 980 nm. Utilizing UCNPs as the energy donor, this method achieves a LOD of 80 particles *μ*L^-1^, significantly minimizing background interference.

### Electrochemical detection

The electrochemical detection method is well-suited for analyzing biological samples due to its high sensitivity, specific target recognition, ease of use, and cost-effectiveness. This approach assesses the properties of targets by monitoring changes in voltage or current. In this process, capture molecules such as antibodies or aptamers are used to bind to the target EVs, while a detection antibody connected to an electroactive signal transducer is utilized to identify the targets and generate electrical signals. The development of electrochemical detection for tumor EVs using aptasensors primarily involves direct immobilization-based detection and label-free detection.

#### Direct immobilization-based detection

Conductive materials are commonly used to modify electrode surfaces, enhancing electron transfer between the electrode and captured EVs. Various techniques are employed to immobilize aptamers onto these modified surfaces. The electrochemical signals generated by the captured EVs can be detected and quantified, providing valuable insights into the presence and concentration of EVs in the sample. Wang *et al.* employed advanced aptamer technology, DNA-based nanostructures, and portable electrochemical devices to develop an aptasensor assisted by nanotetrahedra 139. The precise immobilization of aptamers significantly increased the availability of aptamers containing synthetic nucleobases to bind with suspended EVs, resulting in a 100-fold increase in sensitivity compared to aptasensors modified with individual aptamers. Besides, DNA walkers enhance electrochemical detection by incorporating amplification strategies that increase sensitivity, enabling the detection of EVs at low concentrations. A ratiometric electrochemical biosensor was developed for the direct detection of breast cancer exosomal miR-21, utilizing bipedal DNA walkers powered by locked nucleic acid (LNA) modified toehold mediate strand displacement reaction (TMSDR) 140. The bipedal walker, with its dual pedals, demonstrates superior signal amplification efficiency compared to a single pedal DNA walker, which is prone to drifting away from tracks. Similarly, Zhao *et al.* described a highly sensitive detection method utilizing a target-triggered three-dimensional DNA walking machine and exonuclease III-assisted electrochemical proportional biosensing for the detection of exosomes, achieving a LOD of 13 particles *μ*L^-1^ 141. An exonuclease III-assisted electrochemical ratiometric sensor was employed to further enhance signal amplification. Besides the aforementioned amplification strategies, signal amplification methods based on nanomaterials have also been widely applied. Wu *et al.* described the creation of a nanosensor using a dual-aptamer modified reduced graphene oxide (RGO) field-effect transistor (GFET) platform, adorned with AuNPs 142. This nanosensor achieved a detection limit of 84 particles *μ*L^-1^ for HepG2-MVs. Furthermore, a novel electrochemical biosensor was developed by integrating hierarchical gold nanoarrays onto two-dimensional Ti_2_CT_x_ MXene membranes 143. This biosensor demonstrated a LOD of 58 particles *μ*L^-1^ within a linear range of 1×10^2^ to 1×10^7^ particles *μ*L^-1^.

#### Label-free detection

**Figure 11:**
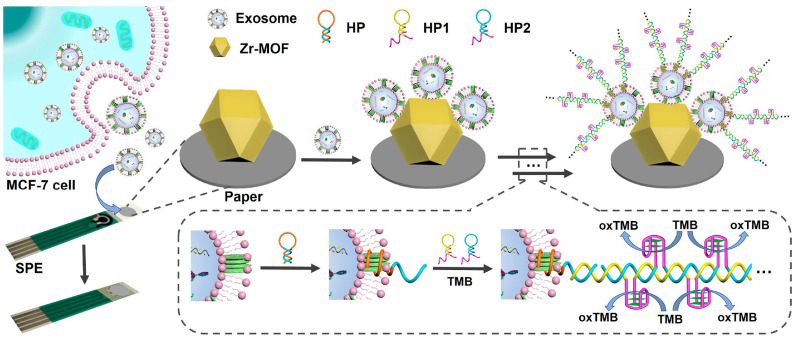
Principle of the paper-based biosensor for exosome assay. Adapted with permission from 145, 2021 American Chemical Society.

Label-free EC detection facilitates the sensitive detection of EVs in samples without requiring electrode biofunctionalization or the labeling of signal tags on aptamers, thereby circumventing complex and costly multistep procedures. Zhang *et al.* developed a straightforward electrochemical aptasensor that operates without labeling, utilizing target-induced proximity hybridization of divided aptamers 144. This sensor captures PTK7-containing EVs using oligonucleotide probes that target PTK7 fragments. In the presence of target EVs, proximity hybridization occurs, leading to the formation of a DNA duplex on the electrode. This duplex subsequently attracts a higher concentration of Ru(NH_3_)_6_^3+^, resulting in an enhanced cathodic current signal. The aptasensor effectively detected tumor-derived EVs with a LOD of approximately 660 particles *μ*L^-1^. In a similar vein, Liu *et al.* engineered a simple electrochemical biosensor that operates without labels by integrating a paper coated with a metal-organic framework (MOF) onto a screen-printed electrode (SPE) 145. This biosensor detects EVs using Zr-MOFs and aptamers, initiating a HCR that generates a DNAzyme for signal amplification, as illustrated in Figure 11. This method surpasses many previously documented techniques, achieving a minimal LOD of 5 particles *μ*L^-1^. A reusable electrochemical biosensor has been developed, which integrates a DNA walker activated by dual-recognition proximity binding using an on-off-on approach 146. The hairpin DNA (H) underwent a conformational change upon hybridizing with intermediate DNA, significantly increasing the distance between the electroactive label and the electrode surface, known as the sensor's "off" state. Exposure to exonuclease led to the degradation of the intermediate DNA and the restoration of the initial hairpin configuration, reactivating the sensor.

### Colorimetric detection

**Figure 12:**
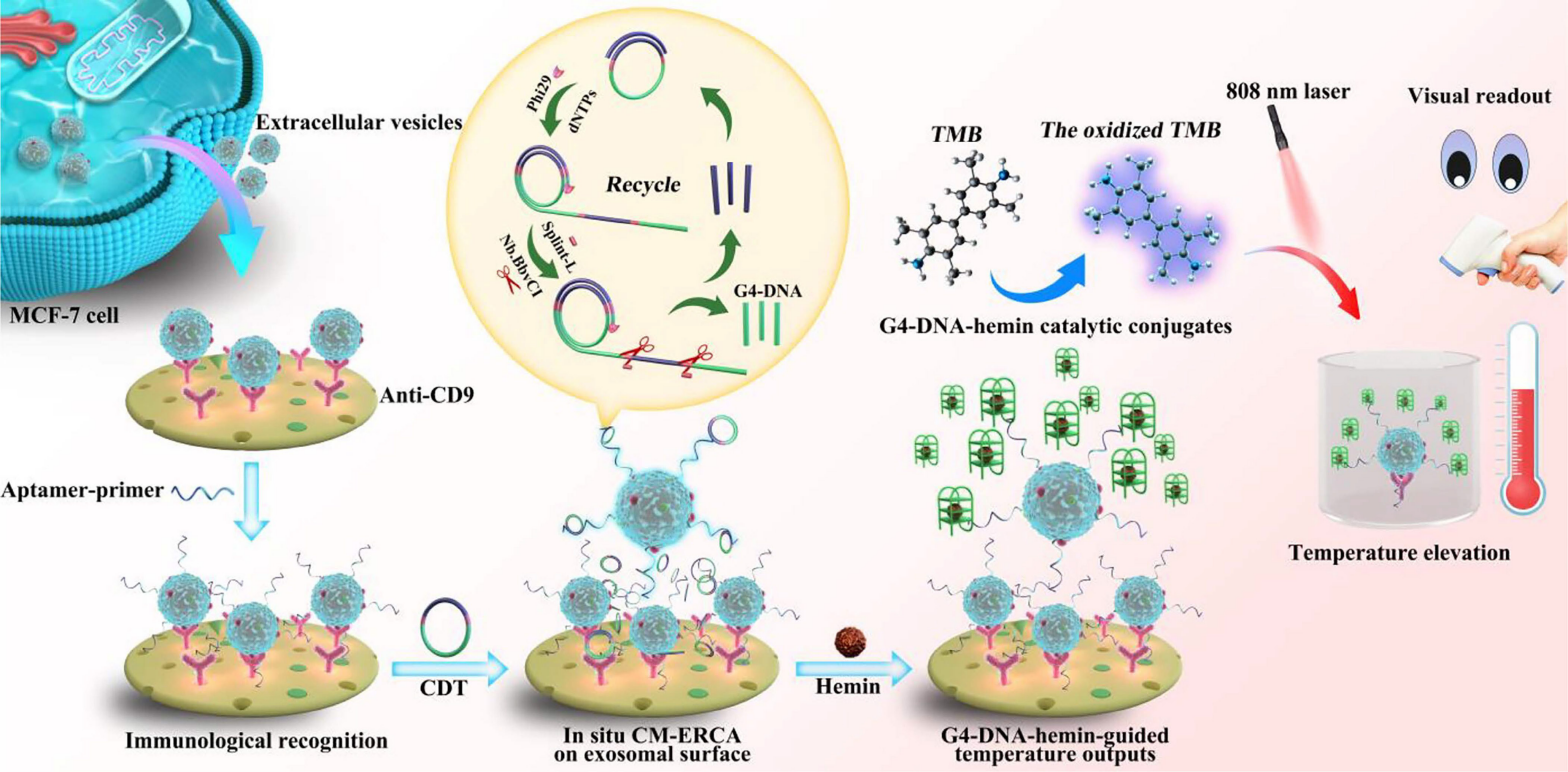
Schematic illustration of the DNA-driven photothermal amplification transducer for highly sensitive visual determination of EVs. Adapted with permission from 154, copyright 2023 American Chemical Society.

Colorimetric methods for EVs detection have garnered significant attention due to their simplicity, cost-effectiveness, and suitability for point-of-care (POC) applications. These methods depend on the color change in a solution or substrate when EVs are present, enabling visual detection without complex instrumentation. The H₂O₂ oxidation reaction, catalyzed by enzymes such as horseradish peroxidase (HRP), involves the oxidation of a chromogenic substrate in the presence of H₂O₂, resulting in a colored product, typically blue or yellow. This product can be quantified using a spectrophotometer or visually compared to a color chart or standard samples. Zou *et al.* reported a colorimetric biosensor for point-of-care testing (POCT) that employs RCA and the H₂O₂ oxidation reaction to detect cancerous EVs 147. In the presence of target EVs, RCA is activated, producing multiple units that bind to DNA probes modified with HRP. This modification causes the transparent ABTS to oxidize, exhibiting strong absorbance in the near-infrared range and enabling both colorimetric and photothermal detection of EVs. Additionally, another colorimetric detection method achieved results within 10 min by catalyzing the transformation of the colorless substrate dopamine into dark-colored polydopamine using HRP in a specially prepared H₂O₂ solution 148. The LOD was significantly improved to 7.7 particle *μ*L^-1^, surpassing conventional dot-blot methods by three to five orders of magnitude. Recently, Wang *et al.* utilized the biotin-streptavidin system combined with MXene nanomaterials to enhance the loading of aptamers and biotin through a dual-effect amplification method, leveraging their substantial specific surface area 149. Other innovative colorimetric biosensors include those based on enzymatic catalysis by glucose oxidase (GOx) 150, 151, G-quadruplex DNAzyme 152-154, and nano-enzymes 155. For instance, Ding *et al.* devised a straightforward platform that uses a DNA-driven photothermal amplification transducer and a simple household thermometer, eliminating the need for sophisticated instruments or labeling processes, as shown in Figure 12 154. The LOD was determined to be 2.7 particle *μ*L^-1^, demonstrating a 10^3^-fold increase in sensitivity compared to fluorescence-based ELISA techniques. Zhang *et al.* developed a highly responsive and versatile visual technique for detecting EVs, which involves silver deposition on Au NRs induced by enzymatic activity and signal amplification via HCR 156. CD63 aptamer-labeled magnetic beads were employed to capture EVs, and the exosomal membrane was modified by integrating cholesterol-modified DNA probes. These probes triggered HCR, leading to increased loading of alkaline phosphatase and the production of ascorbic acid. Subsequently, the ascorbic acid reduced silver ions, resulting in the formation of silver shells on the Au NRs, which shifted the plasmon resonance peak towards the blue spectrum. The method achieved a LOD of 1.6×10^2^ particles *μ*L^-1^ using UV-visible spectroscopy, while visual observation identified limits of 9×10^3^ particles *μ*L^-1^. However, colorimetric methods may exhibit a limited dynamic range, potentially restricting their capacity to accurately quantify a broad spectrum of EVs concentrations, particularly in samples with significant variability in EVs abundance. Overall, colorimetric methods for EVs detection provide a straightforward and rapid means of analysis, though further research and development are necessary to enhance the performance and reliability of these assays.

### Microfluidics integrated detection

**Figure 13:**
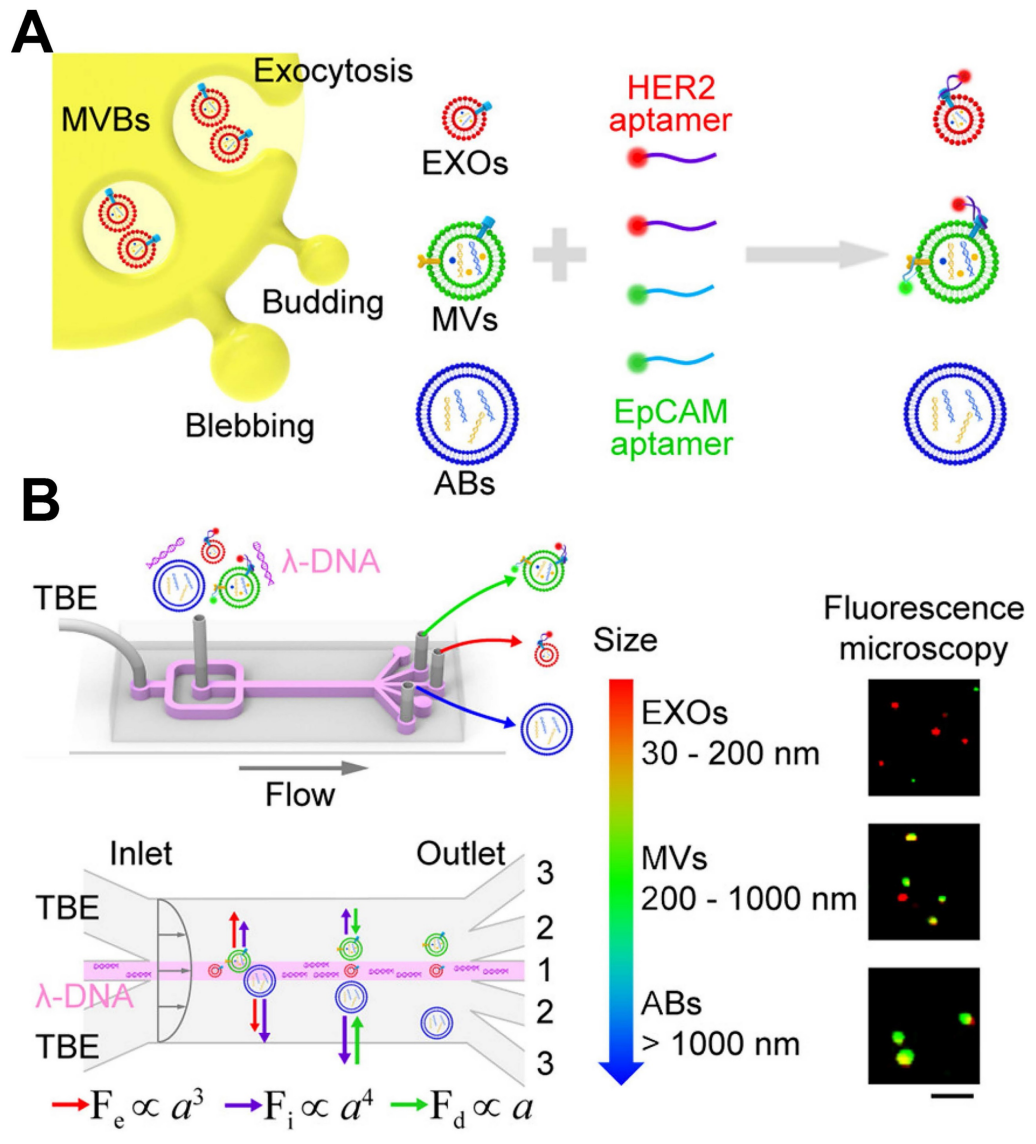
Schematic of λ-DNA-mediated sorting of EV subpopulations and aptamer-based analysis of individual EVs. (**A**) Labeling of cell-originating EVs including exosomes (EXOs, red), microvesicles (MVs, green), and apoptotic bodies (ABs, blue) with fluorescent HER2 and EpCAM aptamers. (**B**) Size-selective separation of EV subpopulations by λ-DNA mediated viscoelastic microfluidics. Fluorescence microscopy images showed HER2 (red) and EpCAM (green) expression of isolated individual EVs. Scale bar, 5 μm. Adapted with permission from 160, copyright 2019 American Chemical Society.

The integration of aptamers with microfluidic devices enables researchers to establish a platform for the capture, isolation, and detection of EVs. Aptamers, immobilized on the surfaces of microfluidic channels or functionalized magnetic beads, facilitate the specific capture of EVs from complex biological samples. Subsequently, the captured EVs can undergo various detection methods for analysis. This integrated approach provides several advantages for EVs detection, including enhanced sensitivity, rapid analysis, and the capacity to analyze small sample volumes. Additionally, it allows for the simultaneous detection of different exosomal markers, enabling the characterization of EVs with specific cargo molecules. The detection of various exosomal miRNAs has been successfully achieved using CHA, which utilizes a hairpin DNA probe and a target RNA molecule to initiate a cascade of signal amplification 157. Exosomal surface proteins and miRNAs were simultaneously detected in situ using a microcolumn array within a ship-shaped microfluidic device. To minimize signal interference, the identification of exosomal surface proteins and miRNAs was conducted in four separate channels using aptamers labeled with Cy5 and a signal amplification technique based on CHA. Zheng *et al.* introduced a novel PTCDI-aptamer signal switch strategy for sensitive quantification, combined with a microfluidic chip for specific isolation of EVs 158. The platform exhibits a wide linear range across six orders of magnitude and a low detection limit of 8.69 particles *μ*L^-1^ for HepG2 EVs, attributed to the effective on-chip enrichment using Tim4-modified magnetic beads. Zhao *et al.* reported a novel method that integrates a functionalized membrane with an automated centrifugal microfluidic disc system to isolate and enrich EVs for clinical applications 159. In clinical sample trials, this approach achieved a diagnostic accuracy of 91% (95% CI 79%-96%), outperforming EVs ELISA for lung cancer detection (area under the curve 0.9378 versus 0.8733, 30 patients). Liu *et al.* conducted an analysis focused on individual EVs, employing λ-DNA and aptamers for the concurrent isolation of distinct EVs according to their size and the examination of surface proteins, as depicted in Figure 13 160. Utilizing a machine learning algorithm, they demonstrated that the isolated microvesicles were superior in differentiating between breast cell lines and Stage II breast cancer patients with varying levels of HER2 expression, through the identification of EVs signatures. In cancer diagnosis, microfluidics offers a promising method for identifying EVs. It provides precise control over fluid flow, enables the integration of various detection strategies, and offers potential for high-throughput analysis. However, further research is necessary to establish standardized detection criteria, enhance machining accuracy, and develop more precise analysis methods for EVs detection based on microfluidics.

### Others

Fluorescence resonance energy transfer (FRET) is a technique used to detect molecular interactions and conformational changes in biological systems, involving energy transfer between a donor and an acceptor fluorophore. The donor fluorophore is attached to an aptamer or antibody that binds to EVs surface markers, while the acceptor fluorophore is linked to a complementary aptamer or antibody. FRET occurs when EVs bind to these labeled probes, inducing a measurable change in the fluorescence signal. Zhang *et al.* reported an innovative self-standard ratiometric FRET nanoprobe, consisting of a Cy3-CD63 aptamer and Ti_3_C_2_ MXene nanosheets 161. The Cy3-CD63 aptamer specifically binds to the Ti_3_C_2_ MXene nanosheets through hydrogen bonds and metal chelate interactions, leading to the quenching of Cy3 fluorescence. The introduction of EVs disrupts this interaction, resulting in the restoration of Cy3 fluorescence. The detection limit for EVs was established at 1.4 particles *μ*L^-1^, which is a thousandfold improvement over traditional ELISA techniques. Recently, Xiao *et al.* introduced a system for detecting extracellular vesicle membrane proteins using FRET signals between aptamer quantum dots and AIEgen dye, eliminating the need for EVs extraction and purification 162. This system demonstrated significantly higher accuracy (100% versus 65%) and sensitivity (100% versus 55%) in lung cancer diagnosis compared to ELISA. Additionally, by incorporating machine learning analysis, the accuracy of early screening was improved from 96.4% to 100% through the profiling of five biomarkers. FRET allows for real-time monitoring of EVs binding, enabling dynamic studies of interactions and uptake, and provides quantitative data on EVs concentration. Furthermore, FRET assays can be integrated with microfluidic devices or biosensors for enhanced high-throughput and multiplexed EVs analysis.

SERS is an optical analytical method that significantly amplifies the Raman scattering signal of molecules adsorbed onto rough metal surfaces or metal nanostructures. This technique has the potential to advance EVs-related diagnostics and treatments in various fields, including cancer research and precision healthcare. Wang *et al.* explored the use of magnetic substrates and SERS probes to detect and characterize EVs 163. They combined capturing substrates with three distinct types of SERS probes, each based on different SERS reporters. The detection of target EVs was indicated by the formation of an apta-immunocomplex, which corresponded to a decrease in the SERS signal in the supernatant. More recently, Cun *et al.* developed a HCR-amplified strategy featuring an Ag-shell nanostructure induced by alkaline phosphatase for the SERS-based assay of EVs 164. Prostate cancer-derived EVs were isolated using magnetic beads modified with prostate-specific membrane antigen aptamers. Following this, the release of the HCR-amplified chain incorporated multiple functional moieties, enhancing the signal. The use of magnetic materials simplified the traditional immunoassay steps, achieving a LOD of 19 particles *μ*L^-1^ within 40 min.

Additionally, Wang *et al.* demonstrated an aptasensor based on SPR facilitated by dual AuNPs 165. Electronic coupling between the Au film and AuNPs was achieved through controlled hybridization, leading to significant signal amplification. This technique also observed coupling effects in plasmonic nanostructures. By employing this method, undesirable binding of AuNPs to the SPR chip surface was effectively prevented, and the SPR sensor's functionality was restored by applying a layer of 11-Mercapto-1-undecanol to the Au film surface.

The phenomenon of Aggregation Induced Emission (AIE) is characterized by the enhanced emission intensity of certain fluorescent molecules during aggregation. This property has been extensively utilized across a variety of sensing and imaging applications. Within the field of EVs detection, AIE-based techniques have been refined to improve the sensitivity and specificity of assays. Utilizing the self-assembly capabilities of imprinted polymer technology, EVs serve as templates to construct EVs-specific three-dimensional cavities on the surfaces of magnetic particles 166. These cavities, formed by the template-eluted imprinted polymers, selectively capture analytes in serum. Then, aptamer-mediated AIE has been employed to selectively activate these captured targets. The effectiveness of this method was evaluated using lysozyme and fuzzy EVs as targets, demonstrating a linear relationship between fluorescence intensity and target concentration in serum, with a notable recovery rate of lysozyme at 107%.

Harnessing the high specificity and sensitivity of droplet digital PCR (ddPCR), Lin *et al.* introduced a novel approach that combines dual-target-specific aptamer recognition, activated upon connection with the EVs membrane, with ddPCR for the precise quantification of tumor-derived exosomal PD-L1 167. This method holds significant potential for transforming EVs into reliable clinical biomarkers and facilitating the exploration of their biological roles.

## Conclusions and future perspectives

We have provided an overview of recent advancements in EVs research, encompassing sample collection, isolation, storage, characterization, analysis, and applications in cancer detection. EVs contain a wealth of biomolecules and cellular information, emerging as promising biomarkers for monitoring various physiological processes and diseases. A significant difference in EVs levels between cancer patients and healthy individuals has been documented. Advances in EVs research have enhanced our understanding of their structural features, fundamental constituents, formation processes, physical attributes, and cellular roles. However, the isolation and preservation of EVs present challenges due to their complex formation, variability, and limited abundance. Although multiple isolation techniques exist, none achieve perfect isolation, with each method having limitations in yield, purity, and specificity. To improve specificity and purity, combining various techniques may be necessary, which could introduce variations in EVs concentration and size. Preservation of EVs generally avoids freezing. The significance of aptasensors in diagnostics and detection technologies is growing, playing an essential role. Several physical principles, including acoustics, thermodynamics, electrodynamics, hydrodynamics, and optics, in conjunction with functional molecules such as aptamers and molecular beacons, and engineering techniques like microfluidics, are utilized to augment the analysis of EVs and their associated biomolecules. Nevertheless, the lack of standardized protocols for the isolation, characterization, and analysis of EVs hinders the comparability of results among various studies. Establishing standardized procedures and reference materials is crucial for progressing EVs research and facilitating its clinical translation.

Aptamer-based EVs detection platforms can be tailored for in-situ detection, enhancing the study of EVs dynamics and interactions within complex biological systems. This capability offers significant opportunities for comprehensive profiling of EVs contents and their associations with disease states. Additionally, aptamers can be engineered to bind specifically to target nucleic acids or proteins, enabling the simultaneous detection of multiple analytes within EVs, which is vital for a range of biological and clinical applications. Despite these benefits, challenges remain that must be overcome to facilitate the widespread adoption of aptamer-based EVs detection. These challenges include the heterogeneous nature and small size of EVs, as well as the complex physical and chemical properties of bodily fluids. Improvements in the efficiency of selective separation-capture and overall analytical capabilities are crucial. Furthermore, identifying markers specific to EVs sources and developing high-performance aptamers for novel and personalized EVs targets are imperative. In contrast, aptamer-based detection methods offer potential for high sensitivity, specificity, accessibility, cost-effectiveness, and user-friendliness. With continuous advancements in research and technology, aptasensors are positioned to significantly contribute to the precise identification, analysis, and clinical application of EVs in precision medicine.

## Figures and Tables

**Table 1:** Summary of sample collection and handling

Sample types	Invasive collection	Storage	Cautions	Reference
Cell culture	No	-20°C (temporarily storage) or -80 °C (long-term storage)	• Using exosome-depleted serum or serum-free media in cell culture helps eliminate interference from exogenous EVs. • Eliminate microbial or viral infections before isolation	31-35
Blood/Serum/Plasma	Yes	-80 °C or in liquid nitrogen	• Use a large diameter needle (such as a 21G needle) to minimize platelet derived EVs • Choose the appropriate anticoagulant (such as sodium citrate or EDTA) • Discard the first 1-2 mL of blood following the puncture • Thawing should be done at 37 °C or room temperature	36-40
Pleural effusions and ascites	Yes	-80 °C	• Maintain a sterile environment to prevent contamination • Collection site is usually between the ribs in the lower back or side of the chest (pleural effusion) or in the lower abdomen (sites)	41-44
Saliva	No	-80 °C	• Abstain from consuming food, beverages, and tobacco for a minimum of one hour before sample collection • Centrifuge or filter to remove bacteria, squamous epithelial cells and food debris before isolation	45-48
Urine	Optional	-80 °C	• Internal reference (such as uromodulin) can be selected to normalize the concentration of EVs in urine • Regulate the diet to avoid the composition of urine can be affected by certain food • It is advised to utilize appropriate protease inhibitors during sample preparation to avoid the deterioration of exosomal proteins while isolation	49-55
Tear fluid	No	-80 °C	• Ensure a clean and sterile environment • Avoid touching the eyelids or ocular surface • Use appropriate collection methods (such as Schirmer test) • Minimize patient discomfort	56-59
Cerebrospinal fluid (CSF)	Yes	-80 °C	• CSF can be collected through ventricular/lumbar drain placement or cisternal aspiration during the craniotomy • Filter the CSF samples using a 0.8 μm filter to remove cellular debris after collection	60-63

**Table 2:** Summary of EVs isolation strategies

Isolation techniques	Principle	Advantages	Disadvantages	Reference
Differential ultracentrifugation	Distinguishing EVs from other components in the sample by their variations in density and size.	• Widely accepted gold standard • Medium purity separation • Cost-effective • Application of various biological samples types and capacity • Compatibility with downstream analysis	• Time-consuming • Low yield • Co-isolation of different EVs subtypes with similar size and density • Equipment and expertise requirements	69
Density gradient ultracentrifugation	Distinction in density between EVs and other particles found in a sample.	• High purity separation • Effectively isolation of individual EVs • Preservation of integrity and biological activity	• Time-consuming • Low yield • Complex procedure • Costly • Potential damage to Evs	70
Ultrafiltration (UF)	EVs in the sample are filtered through the ultrafiltration membrane, with only EVs larger than the membrane's pore size being trapped on the membrane and collected as the retentate.	• Simplicity and ease of use • Time efficiency • Preservation of biological activity • Versatility in EVs size selection • Cost-effectiveness	• Low separation efficiency • Limited purity • Membrane lifespan reduction due to blockage	71
Size exclusion chromatography (SEC)	Molecules or particles are separated according to their size as they pass through a column filled with porous polymer microspheres.	• Preservation of biophysical and functional properties • Application of various biological samples types and capacity • Compatibility with downstream analysis	• Low yield of EVs • Time-consuming process • Potential for sample dilution • Limited scalability	72
Polymer precipitation	Utilization of polymers to generate a hydrophobic setting, inducing the less-soluble constituents of EVs to cluster together and settle out of the solution.	• No specialized equipment required • High Extraction Efficiency • Compatibility with Various Sample Types • Cost-Effective	• Low purity • Low recovery • Uneven particle sizes • Damage to exosomes • Hard-to-remove polymers	73
Immunoaffinity capture	Antibodies specifically recognize the EVs surface markers (CD9, CD63, and CD81) are immobilized onto a solid support (magnetic beads or microfluidic chips).	• High specificity • High purity • Fast and efficient • Compatibility with downstream analysis • Potential for clinical applications	• Limited capture efficiency • Cost and availability of antibodies • Labor-intensive and time-consuming	74
Microfluidic analysis	Based on the principles of microfluidics and the physical properties of EVs, such as size, immunoaffinity, electrical and acoustic properties.	• Beneficial to limited or precious sample • High efficiency • Easily automated • Integration of multiple functions • Cost-effective	• Sample volume limitations • Device complexity	75

**Table 3:** Summary of EVs preservation strategies

Preservation strategies	Advantages	Disadvantages	Reference
Cryopreservation	• Convenient and inexpensive operation • It is suitable for long-term storage • Easy and quick reconstitution	• Could not maintain the integrated structure of EVs (cryoinjury) • Cryoprotectants components may affect biological properties of EVs • Cold-chain transportation is expensive, result in the cryopreservation was not very appropriate for EVs transportation	80-83
Freeze-drying	• Lyophilized EVs is acceptable for preservation and transportation under room temperature • The shelf life of lyophilized EVs can be extend to several years • Reduce the risk of contamination during storage	• Molecular structure may be destroyed by the freezing and dehydration pressures generation • Complex and time-consuming process, requires specialized equipment and expertise • Equipment and materials can be expensive	84-86
Spray-drying	• No freezing procedure • It is available for rapid conversion of EVs solution into a dry powder • It allows for accurate manipulation of the aerodynamic particle size and properties	• The heated gas may lead to damage or disruption of the EVs membrane • The high temperatures and shear forces may lead to aggregation of EVs, resulting in a lower recovery rate	87, 88

**Table 4:** Summary of current EVs characterization methods

Methods	Purpose	Advantages	Disadvantages	Reference
Western blot (WB)	Specific proteins associated with EVs detection and analysis	• High specificity for proteins • Compatibility with large-scale analysis • Established technique	• Large sample volumes requirement • Lengthy workflow • Lack of specificity for Evs • Inability to quantify EVs concentration • Dependence on antibody availability	89
Transmission electron microscopy (TEM)	Observation of the internal structures of EVs	• High resolution • Direct measurement • Surface topography and internal structure information • Avoidance of sample distortion (Cryo-EM)	• Extensive and time-consuming sample preparation procedures • Vacuum requirement • Low throughput • Cost and equipment Requirements • Lack of proteins Characterization	90
Cryo-electron microscopy (Cryo-TEM)	Imaging EVs in natural state	• Preservation of sample integrity • High-resolution • Visualization without staining • Minimal sample distortion	• Expensive equipment • Requirement of cryo-fixation and cryo-sectioning for sample preparation • Ice crystal formation during the freezing process may cause artifacts	91
Atomic-force microscopy (AFM)	Nanoscale imaging and characterization of the morphology, mechanical properties, and biomolecular components of individual EVs	• High-resolution • Native sample conditions • Mechanical properties (stiffness or elasticity) characterization • Real-time imaging	• Sample deposition may result in the loss or alteration of EVs • Time-consuming • Lack of proteins Characterization • Cost and equipment requirements	92
Nanoparticle tracking analysis (NTA)	Determination of the size distribution and concentration of EVs	• Size and concentration determination • Real-time observation • Fast detection speed • High sensitivity	• Difficulty in distinguishing contaminated proteins • Influence of camera levels and detection thresholds • Sample preparation may introduce variability and potential loss of EVs	93
Dynamic light scattering (DLS)	Measurement of hydrodynamic diameter of EVs	• Wide size range measurement • No requirement of labeling or modification of EVs • Rapid analysis • Cost-Effective	• Not suitable for accurately measuring the size distribution of polydisperse vesicles • Difficulty in distinguishing contaminated proteins • Influence of camera levels and detection thresholds	94, 95
Flow cytometry (FCM)	Analysis and characterization of EVs based on physical and biochemical properties	• High-throughput analysis • Single-particle analysis • Multiparametric analysis • Quantitative analysis • Compatibility with other techniques	• Insufficient detection sensitivity and resolution • May not be able to accurately distinguish between individual EVs and aggregates • Time-consuming sample preparation • Background noise • Instrumentation requirements	96

**Table 5:** Summary of EVs detection evaluation

Detection Type	Method	Target	Linear Range (particles μL^-1^)	LOD (particles μL^-1^)	Reference
Fluorescence detection	RCA	CD9, CD63, Calnexin	50 - 1.2×10^3^	42.22	106
		MUC1	1×10^2^ - 1×10^6^	42.7	107
		CD63	0.1 - 1×10^3^	40	108
		CD63, PSA, EpCAM, MUC1, CEA	1×10 - 1×10^5^	9.9	109
		EpCAM	2.5×10^5^ to 1×10^7^	2.5×10^5^	168
	SDR	CD63, MUC1	1.6×10^2^ - 3.2×10^6^, 1×10^2^ - 6.4×10^5^	67, 37	110
		CD63	1×10*^2^* -1×10^6^	1×10^2^	111
		PTK7	5×10^5^ - 7.5×10^7^	3.4×10^5^	112
	HCR	CD63, PTK-7	5×10^2^ - 1×10^7^	4.47×10^2^	113
		CD63, EpCAM or CD63, AFP	1.7×10^4^ - 1.7×10^10^	1.8×10^2^	114
		Nucleolin, PD-L1	64 - 10^6^	1×10^2^	115
		PTK7	1×10^3^ - 1×10^7^	4×10^3^	116
		PD-L1	1 - 1×10^6^	5	169
	CHA	CD63	44 - 8.75×10^5^	26	117
		EpCAM, CD63	8.4 - 8.4×10^5^ for EXO-MCF-7, 9.8 - 9.8×10^4^ for EXO-PANC-1	0.5 for EXO-MCF-7, 0.1 for EXO-PANC-1	118
	Enzymes-assisted	PD-L1	1. ×10^-2^ pg^a^ - 1 pg	5.21×10^-3^ pg	119
		CD9, CD63, CD81, EpCAM, PD-L1	6×10^2^ - 6×10^6^	72	120
		CD63	1×10^2^ - 1×10^6^	1×10^2^	121
		CD109, EGFR	0.1 - 1×10^5^	1×10^2^	122
		CD63	3.6×10^2^ - 7.19×10^6^	360	123
		PD-L1	2.5×10^5^ - 1×10^8^	9.4×10^4^	124
		CD63, VEGF	1.75 - 3.5×10^5^	1.02	170
	Nano-materials-assisted	CD63, EpCAM, CEA, PTK-7, AFP, PSMA, PDGF	1.6×10^2^ - 1.6×10^6^	1.6×10^2^	125
		EpCAM	5×10^2^-2×10^4^	1.86×10^2^	126
		CD63	7.5×10^4^ - 1.5×10^7^	5×10^4^	127
		MUC1	0 - 200	8.56	128
		PD-L1	0.07 - 0.7 ng^a^	2.4×10^-2^ ng^a^	171
	Direct fluorescence detection	CD63	5×10^2^ - 5×10^5^	500	131
		CD63, EpCAM	4.9×10^4^ - 4.9×10^6^	4.9×10^4^	132
Luminescence detection	ECL	EpCAM	5×10^2^ - 5×10^6^	125	133
		CD63	1×10^2^ - 1×10^5^	30	134
		CD63, MUC1	60 - 1×10^5^	36	135
		CD63	3.4×10^2^ - 1.7×10^5^	74.1	136
		CD63	0.95 - 9.5×10^4^	0.641	172
		miR21	100 aM^c^ - 1 nM^c^	4.27 aM^c^	173
	LRET	CD63	1×10^4^ - 1×10^8^	1.1×10^3^	137
		EpCAM	NA^b^	80	138
Electrochemical detection	Direct immobilization	EpCAM	1×10^3^ - 1×10^7^	3.96×10^2^	139
		miR-21	NA	67 aM^c^	140
		CD63, EpCAM	50 - 1×10^7^	13	141
		EpCAM, TLS11a	60 - 6×10^6^	84	142
		EpCAM, CD63	1×10^2^ - 1×10^7^	58	143
		CD63	0.1 - 10^4^	42	174
	Label-free	PTK7	2.375×10^3^ - 9.5×10^5^	6.607×10^2^	144
		CD63	3.4×10^2^ - 3.4×10^5^	5	145
		CD63	50 - 1×10^5^	16	146
Colorimetric detection	H_2_O_2_ oxidation	EpCAM	5×10^3^-10^6^	1×10^3^	147
		CD63, HER2, integrin αvβ6	10 - 1×10^8^	7.77	148
		CD63	1×10^2^ - 1×10^7^	42	149
		MUC1	3.8×10^3^ - 1.2×10^5^	1.51×10^2^	150
		CD63	1.6×10^2^ - 1.6×10^3^	78	151
		MUC1	8.3×10^2^ - 5.3×10^4^	3.94×10^2^	152
		CD63	1×10^3^ - 1×10^7^	267	153
		CD9, CD63	65-1.3×10^5^	2.7	154
		CD63	1.9×10^6^ - 3.38×10^7^	13.52×10^5^	155
	Nano-materials-assisted	CD63	1.4×10^3^ - 2.8×10^5^	1.6×10^2^	156
		CD63	1×10^3^ - 1×10^7^	10^4^	175
Microfluidics integrated detection		EGFR, EpCAM, CD81, CD63	1×10 - 1×10^6^	83	157
		CD63, PD-L1, nucleolin, EpCAM, PTK-7, PSMA	8.74 - 1.37×10^5^	8.74	158
		PD-L1, CA125, CD63, CEA, EpCAM	NA^b^	1.58×10^2^	159
		HER2, EpCAM	NA^b^	Individual EVs	160
		PD-L1	1×10^4^ - 1×10^8^	3.2×10^3^	176
Others	FRET	CD63	10 - 1×10^6^	1.4	161
		CD63, CEA, PD-L1, EpCAM, CA125	67.6 - 5.28×10^6^	1.95	162
	SERS	CD63	32 - 2×10^6^ for SKBR3, 203 - 2×10^6^ for LNCaP, 73 - 2×10^6^ for T84	32 for SKBR3, 203 for LNCaP, 73 for T84	163
		PSMA	1.2×10^2^ to 2.4×10^3^	19	164
	SPR	CD63	NA	5	165
	AIE	NA^b^	790 - 7.9×10^3^	1.3×10^3^	166
	ddPCR	PD-L1	NA	7.35×10^-5^ pg	167

a: the unit is pg when detecting proteins. b: NA, not found. c: the unit is aM or nM when detecting miRNA.

## Abbreviations

ABTS: 2,2'-azinobis-(3-ethylbenzthiazoline-6-sulphonate)
AFM: atomic force microscopy
AuNPs: gold nanoparticles
Au NRs: gold nanorods
CHA: catalytic hairpin assembly
CRISPR: clustered regularly interspaced short palindromic repeats
CSF: cerebrospinal fluid
ddPCR: droplet digital polymerase chain reaction
DLS: dynamic light scattering
DNA: deoxyribonucleic acid
EC: electrochemical
ECL: electrochemiluminescence
EDTA: ethylene diamine tetraacetic acid
ELISA: enzyme-linked immunosorbent assays
EVs: extracellular vesicles
FBS: fetal bovine serum
FCM: flow cytometry
FRET: fluorescence resonance energy transfer
GFET: field-effect transistor
GO: graphene oxide
GOx: glucose oxidase
HCR: hybridization chain reaction
HRP: horseradish peroxidase
ILVs: intraluminal vesicles
ITC: isothermal titration calorimetry
LOD: limit of detection
LRET: luminescence resonance energy transfer
MOF: metal-organic framework
MPA: mercaptopropionic acid
MVBs: multivesicular bodies
MVs: micro vesicles
NTA: nanoparticle tracking analysis
PEG: polyethylene glycol
P-ERCA: probe-based exponential rolling circle amplification
POCT: point-of-care testing
PSMA: prostate-specific membrane antigen
RCA: rolling cycle amplification
RGO: reduced graphene oxide
RNA: ribonucleic acid
SDR: strand displacement reaction
SEC: size exclusion chromatography
SELEX: systematic evolution of ligands by exponential enrichment
SERS: surface-enhanced Raman scattering
SPE: screen-printed electrode
SPR: surface plasmon resonance
TAMRA: tetramethyl rhodamine
TEM: transmission electron microscopy
UCNPs: upconversion nanoparticles
UF: ultrafiltration
UV: ultraviolet
WB: western blot
